# In Utero ART Exposure and Birth and Early Growth Outcomes Among HIV-Exposed Uninfected Infants Attending Immunization Services: Results From National PMTCT Surveillance, South Africa

**DOI:** 10.1093/ofid/ofx187

**Published:** 2017-08-30

**Authors:** Vundli Ramokolo, Ameena E Goga, Carl Lombard, Tanya Doherty, Debra J Jackson, Ingunn MS Engebretsen

**Affiliations:** 1 Health Systems Research Unit, South African Medical Research Council, Cape Town,South Africa; 2 Centre for International Health, Department of Global Public Health and Primary Health Care, University of Bergen, Bergen, Norway; 3 Department of Paediatrics and Child Health, Kalafong Hospital, University of Pretoria, South Africa; 4 Biostatistics Unit, South African Medical Research Council, Cape Town,South Africa; 5 School of Public Health, University of the Western Cape, Cape Town,South Africa; 6 UNICEF, New York, New York

**Keywords:** antiretroviral therapy, birth outcome, HIV, South Africa

## Abstract

**Background:**

Despite the recognized benefit of antiretroviral therapy (ART) for preventing and treating HIV, some studies have reported adverse birth outcomes with in utero ART exposure. We evaluated the effect of infant in utero HIV and ART exposure on preterm delivery (PTD), low birth weight (LBW), small for gestational age (SGA), and underweight for age (UFA) at 6 weeks.

**Methods:**

We surveyed 6179 HIV-unexposed-uninfected (HUU) and 2599 HIV-exposed-uninfected (HEU) infants. HEU infants were stratified into 3 groups: ART, Zidovudine alone, and no antiretrovirals (None). The ART group was further stratified to explore pre- or postconception exposure. Multivariable logistic regression evaluated effects of HIV and ARV exposure on the outcomes.

**Results:**

We found higher odds of PTD, LBW, SGA, and UFA in HEU than HUU infants. HEU in the None group (adjusted odds ratio [AOR], 1.9; 95% confidence interval [CI], 1.2–3.0) or those whose mothers initiated ART preconception (AOR, 1.7; 95% CI, 1.1–2.5) had almost twice the odds of PTD than infants whose mothers started ART postconception, but no increased odds for other outcomes.

**Conclusions:**

There was an association between preconception ART and PTD. As ART access increases, pregnancy registers or similar surveillance should be in place to monitor outcomes to inform future policy.

With the success of the Prevention of Mother To Child HIV Transmission (PMTCT) program, HIV-exposed-uninfected (HEU) children now constitute the majority of the children affected by the HIV pandemic [1]. In January 2015, South Africa (SA), which has a antenatal HIV prevalence of 29%, adopted the 2013 World Health Organization (WHO) recommendation of lifelong antiretroviral treatment (ART) for all HIV-positive pregnant women, referred to as WHO-PMTCT Option-B+, as a PMTCT strategy to maintain maternal health [2, 3]. Although the benefits of ART outweigh the potential adverse effects, this population of HEU children requires monitoring to better understand the short- and long-term health effects of HIV and ART exposure [4]. The shift to Option-B+, and the possible future use of pre-exposure prophylaxis (PreP) as a primary prevention method in HIV-uninfected adolescents and young women [5, 6], will increase the number of children exposed to ART during critical intrauterine, intrapartum, and postpartum periods. It is well established that HIV infection in children is associated with poor birth [7] and postnatal [8–10] outcomes. However, the effect of exposure to maternal HIV infection and ART use on the birth outcomes of HEU infants in resource-limited settings, where HEU children have a higher mortality risk than HUU children [11] and where endemic undernutrition and co-infections can compound any potential adverse effect of HIV or ART exposure, is less clear. Using data from a nationally representative facility-based PMTCT survey, we studied the effect of infant in utero HIV and ART exposure on preterm delivery (PTD), low birth weight (LBW), small for gestational age (SGA) at birth, and underweight for age (UFA) at 6 weeks postpartum.

## METHODS

### Study Design

The 2012–13 South African PMTCT Evaluation (SAPMTCTE) was a nationally representative facility-based cross-sectional survey, conducted between October 2012 and May 2013, to measure vertical HIV transmission at 4–8 weeks postpartum. During this study, ART use was criteria-led (WHO PMTCT “Option-A”), changing to “universal test and treat” for all HIV-positive pregnant women throughout breastfeeding (“Option-B”) in April 2013. Under Option-A, antiretroviral drug (ARV)-naïve HIV-infected pregnant women were placed on ART (recommended tenofovir disoproxil fumarate [TDF] + [3TC]/Emtracitaine [FTC] + Nevirapine [NVP]) if CD4 cell count ≤350 cells/mm^3^ or Zidovudine (ZDV) from 14 weeks gestation (with infant NVP for 6 weeks or until 1 week postbreastfeeding) if CD4 >350 cells/mm^3^ [12].

The study methods have been described elsewhere [13] and are summarized in the Supplementary Materials. In brief, 580 primary health facilities offering immunization services were sampled using a probability proportional to size approach. Consenting mother-infant pairs attending immunization services were consecutively or systematically enrolled, regardless of maternal HIV status, in each facility. Sick infants needing emergency care and those aged <4 weeks or >8 weeks were excluded.

### Exposure Measures

Maternal HIV infection and ART use were the primary exposures. Trained nurse data collectors drew infant dried blood spot (iDBS) specimens during the study visits. All iDBS received HIV antibody (serological) testing, and antibody-positive samples or samples from self-reporting HIV-positive mothers were tested for both HIV-1 proviral DNA and HIV-1 RNA (see the Supplementary Methods). Data collectors used electronic questionnaires to gather self-reported data on the mother’s drug use and timing of initiation. As no maternal blood specimens were collected, a mother was defined as HIV-negative if the infant’s antibody result was negative and HIV-infected if 2 infant HIV antibody test results were positive. An infant was defined as HEU if (1) the HIV antibody result was positive and polymerase chain reaction (PCR) result was negative or (2) the antibody result was positive and PCR equivocal or rejected (1% of sample), or HUU if both results were negative. Among HIV-infected mothers, self-reported ARV use was categorized into 3 groups: namely (1) ART use primarily for mother’s health (ART-group), as per Option-A guidelines, (2) antenatal ZDV as MTCT prophylaxis (ZDV-group), and (3) no ARV use antenatally (None group). Given that ART, particularly TDF, which has been associated with poor birth outcomes, has low bioavailability in breastmilk [14], infants whose mothers only started ART postnatally were excluded from this analysis. In an effort to (1) make the periods of exposure to ARV drugs comparable between women on ZDV versus ART and (2) compare outcomes by duration of ART exposure, women on ART were further dichotomised by ART duration, and those who initiated ART postconception were treated as the reference.

### Outcomes

The outcomes of interest were PTD, LBW, SGA at birth, and UFA at 6 weeks postpartum. Birth weight and length, 6-week weight and length, and gestational age were extracted from the infants’ routine road to health booklets at 4–8 weeks (median, 6 weeks) postpartum. The anthropometric measurements were conducted by routine health facility staff using facility procedures and equipment. Length data were excluded in this analysis due to measurement errors and missing data. Health facility staff routinely estimated infant gestational age at delivery using the last menstrual period (LMP). PTD was defined as birth before 37 completed weeks gestation, LBW as birthweight <2.5 Kg, and SGA as birthweight-for-gestational-age z score below –1.28 (equivalent to <10th percentile) [15, 16]. We estimated birthweight-for-gestational-age z scores using recently published international Intergrowth-21st standards for assessing newborn size for term- and preterm-born infants [17] and LMS growth [18]. We estimated weight-for-age z scores (WAZ) in infants age 4–8 weeks using the WHO growth standards [19] and considered infants to be UFA if their WAZ was below –2 [20].

### Data Cleaning

Anthropometric measurements and z scores were flagged for verification if any of the following criteria were met: birthweight-for-gestational z score <–6 or >6; WAZ less than –6 or >5. Except for gestational age, which had 1% observations set to missing after verification (including 3 gestational ages outside of the range for the Intergrowth standards [20–23 weeks]), the remaining measurements and z scores had <1% observations omitted.

### Covariates

Covariates in the models were selected a priori based on the literature [21] and a conceptual framework (Supplementary Figure 1). Participants were defined as food insecure if they ever ran out of food in the previous year. Multiple correspondence analysis (MCA) was used to to construct the socio-economic status (SES) index (see the Supplementary Materials). Infant feeding practices were established through 8-day recall infant feeding questions, and infants were categorized into 2 groups: (1) “breastfed” if they received any breastmilk and (2) “non-breastfed” if they received no breastmilk. As SA has historical racial inequalities, race was included in the models as a potential social determinant of the study outcomes [22]. Based on the reported race, study infants were classified as (1) “black,” (2) “colored,” a multiracial group, or (3) “other,” comprised of very small samples of infants defined as “white,” “indian,” and “other.”

### Statistical Analysis

Analyses were survey based, and additional weighting for missing gestational age data was applied to the PTD and SGA analyses (see the Supplementary Materials). Categorical variables were compared using the Pearson chi-square test while linear regression was used to test equality of means. The Wald test was used for multiple hypothesis testing. In the modeling, we first generated 4 multivariable logistic regression models to assess the effect of in utero HIV exposure on outcomes in the total sample of HUU and HEU infants. We then restricted the analysis to HEU infants and compared their outcomes by ART exposure status and duration using 4 additional models. To avoid bias introduced by adjustment of potential mediators in the presence of unmeasured common causes, the “LBW paradox” [23], variables such as birthweight were not included in the UFA models. In each full model, we also included interaction terms to test whether infant HIV exposure modifies the effect of other covariates on birth outcomes. Statistical analyses were performed at a 5% significance level using STATA-14 (Stata Corp., College Station, Texas), R Software-3.1.2, and IBM-SPSS Statistics-22 (SPSS Inc, Chicago, Illinois).

### Ethical Considerations

Ethical approval was obtained from the South African Medical Research Council and the Office of Associate Director of Science at the US Centers for Disease Control and Prevention. All participants provided informed consent.

## RESULTS

**Figure 1.**
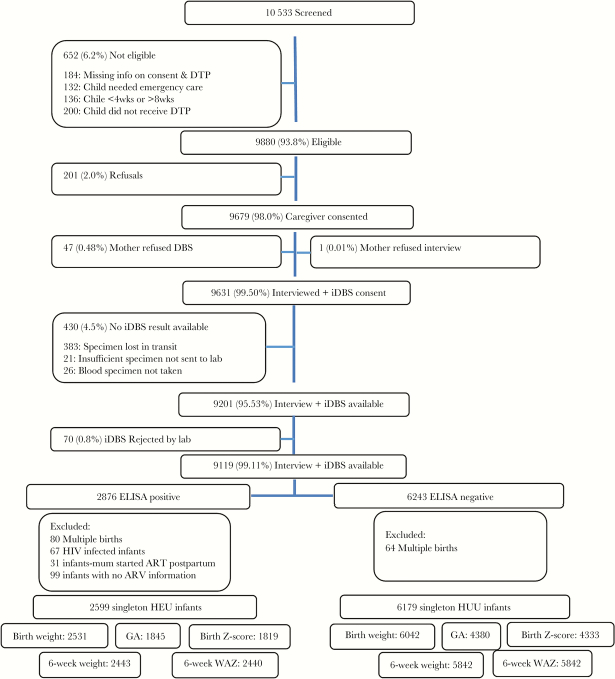
The 2012–13 South African Prevention of Mother To Child HIV Transmission Evaluation study profile. Abbreviations: ART, antiretroviral treatment; ARV, antiretroviral drug; DTP, diphtheria, tetanus and pertussis; ELISA, enzyme-linked immunosorbent assay; GA, gestational age; HEU, HIV exposed uninfected infants; HUU, HIV unexposed infants; iDBS, infant dried blood spot; WAZ, weight-for-age z score.

There were 9119 live-born infants in the SAPMTCTE study, of which 8975 were of a singleton birth (Figure 1). The final sample analyzed included 6179 HUU and 2599 HEU infants. Notably, women who initiated ART preconception were older, had a higher parity, and higher frequencies of TB and syphilis than women in the other ARV groups (Table 1).

**Table 1.** Weighted Analysis of Participant Characteristics by HIV and Antiretroviral Exposure Status

Characteristics	HUU (n = 6179), % (95% CI)	HEU (n = 2599)				Total (n = 8778), % (95% CI)
		Preconception ART (n = 616), % (95% CI)	Postconception ART (n = 780), % (95% CI)	ZDV (n = 873), % (95% CI)	None (n = 330), % (95% CI)	
Maternal						
Age at enrollment, y						
<20	18.3 (17.1–19.6)	1.8 (1.0–3.2)	3.1 (2.0–4.9)	7.0 (5.0–9.6)	5.2 (3.2–8.4)	14.0 (13.1–15.0)
20–25	39.6 (38.2–41.1)	10.0 (7.8–12.8)	28.5 (25.2–32.0)	36.2 (32.6–40.0)	34.9 (29.0–41.3)	35.9 (34.7–37.1)
26–29	17.9 (16.8–19.0)	23.3 (19.7–27.2)	27.2 (24.1–30.5)	26.1 (23.1–29.4)	24.5 (19.7–30.1)	20.3 (19.3–21.3)
30–35	14.0 (12.9–15.1)	35.2 (31.1–39.4)	29.8 (26.5–33.3)	19.1 (16.5–22.0)	20.7 (16.2–26.0)	17.5 (16.5–18.5)
>35	10.0 (9.1–10.9)	29.8 (26.5–33.3)	14.6 (12.0–17.7)	11.6 (9.5–14.0)	14.7 (10.8–19.7)	12.2 (11.4–13.0)
Missing	0.3 (0.2–0.5)	0	0	0	0	0.2 (1.0–3.0)
Parity						
1	43.0 (41.7–44.4)	15.4 (12.5–18.8)	24.8 (21.7–28.2)	30.6 (26.7–34.7)	24.1 (19.2–29.7)	37.3 (36.1–38.4)
2–3	44.4 (43.0–45.7)	63.6 (59.5–67.6)	65.0 (61.2–68.6)	58.8 (54.7–62.8)	63.7 (57.6–69.3)	50.0 (48.8–51.1)
4+	8.54 (7.8–9.3)	21.0 (17.6–24.9)	10.2 (8.1–12.9)	10.64 (8.7–13.0)	12.3 (8.9–16.7)	10.0 (9.3–10.7)
Missing	4.1 (3.5–4.7)	0	0	0	0	2.8 (2.4–3.3)
Education (grade)						
≤grade 7	11.0 (10.0–12.2)	16.9 (14.8–19.2)	15.1 (12.6–17.9)	21.8 (17.3–27.1)	22.1 (15.0–31.3)	12.9 (11.9–14.0)
>grade 7	88.7 (87.5–89.7)	83.1 (80.8–85.2)	85.0 (82.1–87.4)	78.2 (72.9–82.7)	74.5 (64.9–82.3)	82.9 (81.1–84.6)
Missing	0.3 (0.2–0.5)	0	0	0	0	0.2 (0.1–0.4)
Delivery mode						
Vaginal	76.8 (75.4–78.1)	71.3 (66.9–75.3)	73.2 (69.3–76.8)	76.8 (73.5–79.9)	78.0 (71.9–83.1)	76.1 (74.7–77.4)
C-section	23.1 (21.8–24.4)	28.7 (24.7–33.1)	26.5 (22.9–30.4)	23.2 (20.1–26.5)	21.9 (16.8–28.0)	23.8 (22.5–25.1)
Missing	0.2 (0.1–0.3)	0	0.3 (0.1–1.1)	0	0.1 (0.0–0.8)	1.5 (0.8–2.8)
ANC visits						
1–5	44.6 (42.2–47.2)	41.9 (40.0–47.0)	38.0 (32.8–43.5)	39.2 (35.2–43.3)	48.4 (42.3–54.6)	43.4 (41.0–45.8)
5+	23.4 (21.1–25.8)	31.7 (26.6–37.3)	28.7 (24.4–33.5)	26.91(23.1–31.1)	21.7 (16.8–27.6)	24.8 (22.5–27.2)
Missing	32.0 (29.2–34.9)	26.4 (21.8–31.5)	33.3 (27.6–39.4)	33.9 (28.7–39.6)	29.9 (24.0–36.5)	31.8 (29.0–43.8)
Syphilis during pregnancy						
Positive	1.1 (0.8–1.5)	8.1 (4.8–13.1)	7.6 (4.6–12.2)	4.6 (2.9–7.1)	2.6 (1.3–5.1)	2.7 (2.0–3.6)
Negative	68.3 (65.5–71.0)	64.9 (59.0–70.4)	66.8 (61.3–71.8)	70.4 (66.4–74.2)	58.1 (51.4–64.5)	67.8 (65.1–70.3)
Missing	30.6 (28.0–33.4)	27.1 (22.2–32.6)	25.7 (21.4–30.4)	25.0 (21.4–28.9)	39.24 (32.89–46.0)	29.6 (27.1–32.2)
Tuberculosis during pregnancy						
Yes	1.3 (1.0–1.6)	9.5 (7.1–12.5)	5.1 (3.4–7.5)	2.2 (1.2–4.1)	1.89 (0.75–4.73)	2.4 (2.0–2.8)
No	94.5 (93.7–95.2)	90.0 (87.0–92.4)	94.4 (91.7–96.2)	97.6 (95.7–98.7)	97.88 (95.07–99.10)	94.6 (93.9–95.2)
Missing	4.2 (3.7–4.9)	0.5 (0.1–1.6)	0.5 (0.2–1.4)	0.1 (0.0–0.7)	0.23 (0.03–1.61)	3.0 (2.6–3.5)
No food	14.0 (12.4–15.7)			18.6 (15.4–22.2)	14.9 (10.4–20.8)	
Household socio- economic quintile						
Poorest	15.2 (13.5–17.0)	16.5 (13.4–20.2)	15.1 (12.4–18.2)	16.3 (13.5–19.6)	21.6 (17.0–27.0)	15.6 (14.0–17.4)
Second	16.9 (15.4–18.6)	17.6 (14.3–21.5)	21.0 (17.8–24.7)	20.9 (17.8–24.3)	18.0 (13.9–23.0)	17.8 (16.3–19.4)
Third	17.8 (16.4–19.3)	20.5 (17.2–24.2)	21.3 (17.9–25.1)	23.0 (19.4–27.0)	24.0 (19.4–29.3)	19.1 (17.8–20.5)
Fourth	35.8 (33.5–38.1)	35.2 (30.9–39.7)	34.1 (29.6–35.2)	30.6 (26.3–35.2)	28.1 (22.9–33.9)	34.7 (32.6–37.0)
Least poor	14.3 (12.8–16.0)	10.2 (7.5–13.8)	8.6 (6.5–11.2)	9.3 (7.0–12.2)	8.4 (4.6–14.7)	12.7 (11.4–14.2)
Missing	0	0	0	0	0	0
Infant						
Male	51.4 (50.0–52.7)	50.5 (46.2–54.8)	48.5 (44.2–52.8)	54.8 (51.2–58.4)	51.8 (46.1–57.4)	51.4 (50.3–52.6)
Race						
Black	85.7 (83.0–88.1)	97.3 (94.9–98.5)	98.2 (97.1–99.0)	96.2 (94.1–97.5)	97.5 (94.5–98.9)	89.3 (87.2–91.1)
Colored	12.6 (10.4–15.2)	2.7 (1.5–5.1)	1.8 (1.0–3.0)	3.3 (2.0–5.3)	2.0 (0.9–4.7)	9.5 (7.8–11.5)
Other	1.7 (1.1–2.6)	0	0	0.6 (0.2–1.9)	0.5 (0.1–3.5)	1.3 (0.8–1.9)
Breast feeding						
Yes	88.0 (86.9–89.1)	58.2 (52.5–63.6)	62.8 (59.1–66.4)	65.7 (62.2–69.0)	73.0 (66.7–78.6)	80.6 (79.3–81.8)
No	12.0 (10.9–13.1)	41.8 (36.4–47.5)	37.2 (33.6–40.9)	34.3 (31.0–37.8)	27.0 (21.4–33.3)	19.4 (18.2–20.7)

Abbreviations: ART: antiretroviral treatment; CI: confidence interval; HEU: HIV-exposed uninfected infants; HUU: HIV-unexposed infants; LBW: low birthweight; None: infant was not exposed to in utero antiretroviral drugs; PTD: preterm delivery; SGA: small for gestational age; UFA: underweight for age; ZDV: Zidovudine prophylaxis.

### Prevalence of PTD, LBW, SGA, and 6-Week Childhood Underweight

The prevalences of PTD, LBW, and SGA were 12.5% (95% confidence interval [CI], 11.4–13.7%), 10.7% (95% CI, 10.0–11.5%), and 14.9% (95% CI, 13.8–16.1%), respectively, in the total sample (Table 2). The prevalence of PTD was similar between HUU (12.3%; 95% CI, 11.1–13.7%) and HEU (12.9%; 95% CI, 11.12–14.7%) infants (*P* = .59) in the unadjusted analysis, whereas the latter group had a higher prevalence of LBW (13.0% vs 9.8%; *P* < .01) and SGA (16.9% vs 14.0%; *P* = .03). UFA at 6 weeks postpartum was observed in 9.2% (8.6%; 10.0%) of the total sample. A higher proportion of infants were underweight in HEU (11.0%) versus HUU (8.4%) infants (*P* < .01).

**Table 2.** Adverse Birth and Growth Outcomes by HIV and ARV Exposure Status

Group		PTD, n [% (95% CI)]	LBW, n [% (95% CI)]	SGA, n [% (95% CI)]	UFA, n [% (95% CI)]
HUU	n **=** 6179	568 [12.3 (11.1–13.7)]	624 [9.8 (8.9–10.7)]	590 [14.0 (12.8–15.4)]	531 [8.44 (7.7–9.29)]
HEU	Preconception ART n **=** 616	67 [14.6 (11.6–18.3)]	84 [12.9 (10.4–15.9)]	79 [18.2 (14.7–22.3)]	74 [11.1 (8.8–13.9)]
	Postconception ART n **=** 780	61 [9.5 (7.1–12.5)]	111 [13.7 (11.3–16.5)]	106 [19.2 (15.5–23.5)]	82 [9.9 (7.9–12.4)]
	ZDV n **=** 873	84 [13.0 (10.4–16.2)]	93 [11.3 (9.1–13.9)]	90 [14.8 (11.9–18.3)]	91 [11.1 (8.9–13.7)]
	None n **=** 330	39 [18.2 (13.2–24.6)]	51 [16.1 (12.5–20.5)]	30 [13.9 (9.4–20.0)]	43 [13.9 (10.5–18.2)]
	Total N **=** 2599	251 [12.9 (11.2–14.7)]	339 [13.0 (11.6–14.4)]	305 [16.9 (14.7–19.2)]	290 [11.0 (9.9–12.4)]
Grand total	N **=** 8778	819 [12.5 (11.4–13.7)]	963 [10.7 (10.0–11.5)]	895 [14.9 (13.8–16.1)]	821 [9.2 (8.6–10.0)]

Two hundred five (1.7%) participants had missing data for LBW, 2553 (26.1%) for PTD and 2626 (26.8%) for SGA.

Abbreviations: ART; antiretroviral treatment, low birthweight; CI: confidence interval; LBW, low birth weight; HEU, HIV-exposed uninfected infants; HUU, HIV-unexposed infants; None, infant was not exposed to in utero antiretroviral drugs; PTD, preterm delivery; SGA, small for gestational age; UFA, underweight for age; ZDV; Zidovudine prophylaxis

### Factors Related to PTD, LBW, and SGA

Multivariable analyses demonstrated a higher odds of PTD if the infant was HEU versus HUU (adjusted odds ratio [aOR], 1.2; 95% CI, 1.0–1.5), was of colored versus black race (aOR, 1.7; 95% CI, 1.3–2.3), was born to a mother with less than a secondary education (aOR, 1.4; 95% CI, 1.1–1.7), and resided in a poorer household (aOR, 1.7; 95% CI, 1.2–2.5). There was a reduced odds of PTD if the infant was born to a mother age 30–35 years versus <20 years (aOR, 0.7; 95% CI, 0.5–0.9) and had more than 5 antenatal care (ANC) visits versus ≤5 (aOR, 0.7; 95% CI, 0.6–0.9) (Table 3). Within the HEU subgroup, infants in the None group (aOR, 1.9; 95% CI, 1.1–3.1) or those whose mothers initiated ART preconception (aOR, 1.7; 95% CI, 1.1–2.5) had almost twice the odds of PTD than infants whose mothers initiated ART postconception.

**Table 3.** Weighted Multivariable Logistic Regression Models for Factors Related to Preterm Delivery in (A) HIV-Exposed-Uninfected and HIV-Unexposed-Uninfected Infants combined and (B) HIV-Exposed-Uninfected Infants only^a^

Variable	(A) HEU and HUU^b^ Combined		(B) HEU Only^c^	
	aOR (95% CI)	*P* Value^d^	aOR (95% CI)	*P* Value
HIV exposure status				
HEU	1.2 (1.0–1.5)	.04	----	----
HUU	Ref			
ARV	----			.04^*^
Preconception ART			1.7 (1.1–2.5)	.02
ZDV			1.4 (0.9–2.0)	.11
None			1.9 (1.1–3.1)	.01
Postconception ART			Ref	
Syphilis serology				
Positive	0.7 (0.4–1.2)	.22	0.7 (0.3–1.3)	.22
Negative	Ref		Ref	
Tuberculosis				
Yes	1.1 (0.7–1.9)	.65	1.1 (0.5–2.3)	.83
No	Ref		Ref	
Maternal age, years		.08^*^		.08^*^
20–25	0.9 (0.7–1.1)	.36	1.5 (0.7–3.2)	.31
26–29	0.9 (0.7–1.3)	.63	1.5 (0.7–3.5)	.35
30–35	0.7 (0.5–0.9)	.02	1.0 (0.5–2.3)	.94
>35	1.0 (0.6–1.4)	.79	2.0 (0.9–4.6)	.11
<20	Ref		Ref	
Parity		.20^*^		.08^*^
2–3	0.8 (0.7–1.0)	.06	0.7 (0.4–0.9)	.02
4+	0.9 (0.6–1.2)	.40	0.7 (0.4–1.2)	.14
1	Ref		Ref	
Maternal education				
≤ grade 7	1.4 (1.1–1.7)	<.01	1.1 (0.8–1.6)	.62
> grade 7	Ref		Ref	
ANC visits				
+5	0.7 (0.6–0.9)	<.01	0.7 (0.5–1.0)	.08
1–5	Ref		Ref	
Household socio-economic quintile		<.01^*^		.02^*^
Poorest	1.7 (1.2–2.5)	<.01	3.0 (1.3–6.8)	<.01
Second	1.2 (0.9–1.7)	.26	1.7 (0.7–3.8)	.24
Third	1.3 (1.0–1.8)	1.00	2.6 (1.2–5.9)	.02
Fourth	1.1 (0.8–1.5)	.68	2.1 (0.9–4.4)	.07
Least poor	Ref		Ref	
Household food insecurity				
Yes	0.8 (0.6–1.0)	.07	1.0 (0.6–1.5)	.82
No	Ref		Ref	
Infant race		<.01^*^		.41^*^
Colored	1.7 (1.3–2.3)	<.01	1.4 (0.7–2.7)	
Other	1.3 (0.5–3.4)	.56	----	
Black	Ref		Ref	
Infant gender				
Male	0.9 (0.8–1.0)	.12	1.1 (0.8–1.5)	.55
Female	Ref		Ref	

Abbreviations: ANC, antenatal care; aOR, adjusted odds ratio; ART, antiretroviral therapy; ARV, antiretroviral drug; CI, confidence interval; HEU, HIV-exposed-uninfected; HUU, HIV-unexposed; Ref, reference category; ZDV, Zidovudine.

^a^The values in the models are aOR (95% CI).

bModel included 6214 HIV-exposed and -unexposed infants.

cModel included 1839 HIV-exposed-uninfected infants.

dExcept for the asterisk below, the *P* values in this table are *t* test *P* values. The 5% significance level was used in all analyses.

^*^This *P* value is derived from the joint hypothesis testing adjusted Wald test.

A higher odds of LBW was observed if an infant was HEU versus HUU (aOR, 1.6; 95% CI, 1.3–1.9), born to a mother with TB during pregnancy (aOR, 1.6; 95% CI, 1.1–2.5), and if the infant was of colored versus black race (aOR, 2.0; 95% CI, 1.5–2.6), was born to a mother with less than a secondary education (aOR, 1.3; 95% CI, 1.0–1.6), and resided in a poorer household (aOR, 1.5; 95% CI, 1.1–2.0); a reduced odds of LBW was observed if the infant was born to an older mother versus a mother age <20 years (aOR, 0.6; 95% CI, 0.5–0.8), a mother who had more than 5 versus ≤5 ANC visits (aOR, 0.8; 95% CI, 0.6–0.9), or if the infant was male versus female (aOR, 0.8; 95% CI, 0.7–0.9) (Table 4).

**Table 4.** Weighted Multivariable Logistic Regression Models for Factors Related to Low Birth Weight in (A) HIV-Exposed-Uninfected and HIV-Unexposed-Uninfected Infants combined and (B) HIV-Exposed-Uninfected Infants only^a^

Variable	(A) HEU and HUU Combined^b^		(B) HEU Only^c^	
	aOR (95% CI)	*P* Value^d^	aOR (95% CI)	*P* Value
HIV exposure status				
HEU	1.6 (1.3–1.9)	<.01	----	
HUU	Ref			
ARV	----			.27^*^
Preconception ART	----		0.9 (0.6–1.3)	.54
ZDV			0.8 (0.6–1.1)	.14
None			1.1 (0.8–1.6)	.47
Postconception ART			Ref	
Syphilis serology				
Positive	0.8 (0.5–1.3)	.29	0.6 (0.3–1.2)	.15
Negative	Ref		Ref	
Tuberculosis				
Yes	1.6 (1.0–2.5)	.03	1.3 (0.7–2.6)	.46
No	Ref		Ref	
Maternal age, years		<.01^*^		.02^*^
20–25	0.8 (0.6–1.0)	.02	1.0 (0.5–1.9)	.95
26–29	0.6 (0.5–0.8)	<.01	0.8 (0.4–1.6)	.59
30–35	0.6 (0.4–0.8)	<.01	0.7 (0.4–1.5)	.38
>35	0.9 (0.6–1.2)	.49	1.5(0.7–3.0)	.28
<20	Ref		Ref	
Parity		.45^*^		.40^*^
2–3	1.0 (0.8–1.2)	.87	0.9 (0.6–1.2)	.46
4+	0.8 (0.6–1.1)	.23	0.7 (0.4–1.2)	.18
1	Ref		Ref	
Maternal education				
≤grade 7	1.3 (1.0–1.6)	.03	1.0 (0.7–1.4)	.98
>grade 7	Ref		Ref	
ANC visits				
+5	0.8 (0.6–0.9)	.01	0.7 (0.5–1.0)	.07
1–5	Ref		Ref	
Household socio-economic quintile		.03^*^		.43^*^
Poorest	1.1 (0.8–1.5)	.66	1.2 (0.6–2.1)	.66
Second	1.2 (0.9–1.7)	.20	1.1 (0.6–2.1)	.78
Third	1.5 (1.1–2.0)	.01	1.5 (0.8–2.8)	.22
Fourth	1.3 (0.9–1.7)	.16	1.4 (0.7–2.6)	.34
Least poor	Ref		Ref	
Household food insecurity				
Yes	1.1 (0.9–1.4)	.22	1.2 (0.9–1.7)	.27
No	Ref		Ref	
Infant race		<.01^*^		
Colored	2.0 (1.5–2.6)	<.01	3.4 (1.8–6.6)	<.01
Other	1.2 (0.5–2.8)	.67	----	
Black	Ref		Ref	
Infant gender				
Male	0.8 (0.7–0.9)	<.01	0.8 (0.6–1.0)	.03
Female	Ref			

Abbreviations: ANC, antenatal care; aOR, adjusted odds ratio; ART, antiretroviral therapy; ARV, antiretroviral drug; CI, confidence interval; HEU, HIV-exposed-uninfected; HUU, HIV-unexposed; Ref, reference category; ZDV, Zidovudine.

^a^The values in the models are aOR (95% CI).

bModel included 8476 HIV-exposed and -unexposed infants.

^c^Model included 2510 HEU infants.

dExcept for the asterisk below, the *P* values in this table are *t* test *P* values. The 5% significance level was used in all analyses.

^*^This *P* value is derived from the joint hypothesis testing adjusted Wald test.

A higher odds of SGA was observed if an infant was HEU versus HUU (aOR, 1.3; 95% CI, 1.1–1.6) and born of colored versus black race (aOR, 1.6; 95% CI, 1.3–2.0). Factors protective against SGA were older maternal age (aged 26–29) versus aged <20 years (aOR, 0.6; 95% CI, 0.4–0.8) and attendance of 5 versus ≤5 ANC visits (aOR, 0.8; 95% CI, 0.7–1.0) (Table 5).

**Table 5.** Weighted Multivariable Logistic Regression Models for Factors Related to Small for Gestational Age in (A) HIV-Exposed-Uninfected and HIV-Unexposed-Uninfected Infants combined and (B) HIV-Exposed-Uninfected Infants only^a^

Variable	(A)HEU and HUU Combined^b^		(B)HEU Only^c^	
	aOR (95% CI)	*P* Value^d^	aOR (95% CI)	*P* Value
HIV exposure status				
HEU	1.3 (1.1–1.6)	<.01	----	
HUU	Ref			
ARV	----			.14^*^
Preconception ART			0.9 (0.6–1.3)	.52
ZDV			0.7 (0.5–1.0)	.05
None			0.7 (0.4–1.1)	.08
Postconception ART			Ref	
Syphilis serology				
Positive	1.3 (0.8–2.3)	.30	1.6 (0.9–2.8)	.15
Negative	Ref		Ref	
Tuberculosis				
Yes	1.3 (0.8–2.1)	.31	1.1 (0.6–2.2)	.76
No	Ref		Ref	
Maternal age, years		<.01^*^		.03^*^
20–25	0.8 (0.6–1.0)	.10	0.8 (0.4–1.5)	.45
26–29	0.6 (0.4–0.8)	<.01	0.6 (0.3–1.2)	.17
30–35	0.7 (0.5–1.0)	.05	0.6 (0.3–1.2)	.15
>35	1.0 (0.7–1.4)	.91	1.2 (0.6–2.5)	.66
<20	Ref		Ref	
Parity		.67^*^		.65^*^
2–3	0.9 (0.8–1.1)	.42	0.9 (0.7–1.3)	.64
4+	0.9 (0.6–1.2)	.46	0.8 (0.5–1.3)	.36
1	Ref		Ref	
Maternal education				
≤grade 7	1.1 (0.9–1.4)	.37	1.4 (1.0–2.0)	.04
>grade 7	Ref		Ref	
ANC visits				
+5	0.8 (0.7–1.0)	.02	0.9 (0.7–1.3)	.69
1–5	Ref		Ref	
Household socio-economic quintile		.35^*^		.65^*^
Poorest	1.3 (1.0–1.8)	.09	1.3 (0.7–2.3)	.41
Second	1.4 (1.0–1.9)	.06	1.2 (0.6–2.3)	.60
Third	1.4(1.0–1.9)	.07	1.3 (0.6–2.4)	.52
Fourth	1.3 (0.9–1.8)	.14	1.0 (0.5–1.9)	.97
Least poor	Ref		Ref	
Household food insecurity				
Yes	1.0 (0.8–1.3)	.87	0.8 (0.6–1.2)	.27
No	Ref		Ref	
Infant race		<.01^*^		
Colored	1.6 (1.3–2.0)	<.01	2.1 (1.0–4.4)	.04
Other	1.5 (0.3–7.4)	.62	----	
Black	Ref		Ref	
Infant gender				
Male	1.0 (0.9–1.2)	.67	0.9 (0.7–1.2)	.60
Female	Ref		Ref	

Abbreviations: ANC, antenatal care; aOR, adjusted odds ratio; ART, antiretroviral therapy; ARV, antiretroviral drug; CI, confidence interval; HEU, HIV-exposed-uninfected; HUU, HIV-unexposed; Ref, reference category; ZDV, Zidovudine.

^a^The values in the models are aOR (95% CI). Only variables that had a significant association with low birth weight in the bivariate analysis were included the final multivariable models.

bModel included 6142 HIV-exposed and -unexposed infants.

cModel included 1813 HIV-exposed-uninfected infants.

dExcept for the asterisk below, the *P* values in this table are *t* test *P* values. The 5% significance level was used in all analyses.

^*^This *P* value is derived from the joint hypothesis testing adjusted Wald test.

### Factors Related to UFA at 6 Weeks Postpartum

A greater odds of UFA at 6 weeks postpartum was observed if an infant was HEU versus HUU (aOR, 1.5; 95% CI, 1.2–1.8), if colored versus black race (aOR, 2.2; 95% CI, 1.6–2.8), was born by caesarean section (C-section) versus vaginal delivery (aOR, 1.4; 95% CI, 1.1–1.7), had experienced diarrheal episodes in the first 6 weeks of life (aOR, 1.9; 95% CI, 1.3–2.8), was born to mother who had TB during pregnancy (aOR, 1.8; 95% CI, 1.2–2.8) or had less than a secondary education (aOR, 1.4; 95% CI, 1.1–1.8) and resided in a poorer household (aOR, 1.5; 95% CI, 1.1–2.0). A reduced odds of UFA was observed if the infant was breastfed versus not breastfed (aOR, 0.8; 95% CI, 0.6–1.0) and if the infant was born to a mother who attended more than 5 ANC visits versus ≤5 (aOR, 0.8; 95% CI, 0.6–1.0) (Table 6). None of the models showed evidence of effect measure modification by HIV exposure status (data not shown).

**Table 6.** Weighted Multivariable Logistic Regression Models for Factors Related to Underweight for Age in (A) HIV-Exposed-Uninfected and HIV-Unexposed-Uninfected Infants combined and (B) HIV-Exposed-Uninfected Infants only^a^

Variable	HEU and HUU Combined^b^		HEU Only^c^	
	aOR (95% CI)	*P* Value^d^	aOR (95% CI)	*P* Value
HIV exposure status			----	
HEU	1.5 (1.2–1.8)	<.01		
HUU	Ref			
ARV				.43^*^
Preconception ART	----		1.1 (0.7–1.6)	.78
ZDV			1.1 (0.8–1.6)	.64
None			1.4 (0.9–2.2)	.12
Postconception ART			Ref	
Syphilis serology				
Positive	0.7 (0.4–1.1)	.12	0.5 (0.3–1.0)	.05
Negative	Ref		Ref	
Tuberculosis				
Yes	1.8 (1.2–2.8)	<.01	1.8 (1.0–3.2)	.04
No	Ref		Ref	
Maternal age, years		.04^*^		.15^*^
20–25	0.8 (0.6–1.0)	.06	0.8 (0.4–1.4)	.34
26–29	0.7 (0.5–0.9)	.01	0.7 (0.4–1.3)	.30
30–35	0.7 (0.5;0.9)	.02	0.7 (0.4–1.3)	.21
>35	0.9 (0.6–1.3)	.48	1.1 (0.6–2.2)	.72
<20	Ref		Ref	
Parity		.33^*^		.62^*^
2–3	0.9 (0.7–1.1)	.15	0.9 (0.6–1.3)	.52
4+	0.8 (0.6–1.2)	.27	0.8 (0.4–1.4)	.33
1	Ref		Ref	
Maternal education				
≤7	1.4 (1.1–1.8)	<.01	1.20 (0.8–1.7)	.32
>7	Ref		Ref	
ANC visits				
+5	0.8 (0.6–1.0)	.02	0.9 (0.6–1.2)	.31
1–5	Ref		Ref	
Household socio- economic quintile		.01^*^		.59^*^
Poorest	1.5 (1.1–2.0)	.02	1.4 (0.8–2.7)	.25
Second	1.2 (0.9–1.7)	.23	1.2 (0.6–2.4)	.58
Third	1.6 (1.2–2.1)	<.01	1.6 (0.8–2.9)	.16
Fourth	1.2 (0.9–1.6)	.32	1.3 (0.7–2.5)	.38
Least poor	Ref		Ref	
Household food insecurity				
Yes	1.2 (0.9–1.5)	.14	1.1 (0.8–1.6)	.63
No	Ref		Ref	
Delivery method				
C-section	1.4 (1.1–1.7)	<.01	1.4 (1.0–1.9)	.03
Vaginal	Ref		Ref	
Infant race		<.01^*^		
Colored	2.2 (1.6–2.8)	<.01	2.6 (1.4–5.0)	<.01
Other	1.3 (0.5–3.5)	.67		----
Black	Ref		Ref	Ref
Infant gender				
Male	1.2 (1.0–1.4)	.04	1.4 (1.0–1.8)	.04
Female	Ref		Ref	Ref
Feeding				
Breastfed	0.8 (0.6–1.0)	.05	1.1 (0.8–1.5)	.56
None breastfed	Ref		Ref	
Diarrhea				
Yes	1.9 (1.3–2.8)	<.01	1.8 (0.8–4.0)	.18
No	Ref		Ref	

Abbreviations: ANC, antenatal care; aOR, adjusted odds ratio; ART, antiretroviral therapy; ARV, antiretroviral drug; CI, confidence interval; HEU, HIV-exposed-uninfected; HUU, HIV-unexposed; Ref, reference category; ZDV, Zidovudine.

^a^The values in the models are aOR (95% CI). Only variables that had a significant association with low birth weight in the bivariate analysis were included the final multivariable models.

bModel included 8202 HIV-exposed and -unexposed infants.

cModel included 2414 HIV-exposed-uninfected infants.

dExcept for the asterisk below, the *P* values in this table are *t* test *P* values. The 5% significance level was used in all analyses.

^*^This *P* value is derived from the joint hypothesis testing adjusted Wald test.

## DISCUSSION

In a nationally representative survey, we observed greater odds of PTD, LBW, SGA, and UFA among HEU than HUU infants.

We observed that HEU infants whose mothers did not receive ARVs carried higher odds of PTD than postconception ART-exposed infants but no increased odds for LBW, SGA, and underweight. In addition, among ART-exposed pregnancies, PTD was more common among infants whose mothers initiated ART preconception than postconception. We also identified several independent risk factors for poor birth and growth outcomes, including colored race, which was associated with all study outcomes; birth by C-section, which was associated with UFA; exposure to maternal TB during pregnancy, which was associated with LBW and UFA; and diarrhea was a risk factor for UFA; a lower maternal education and household SES, which were associated with PTD, LBW, and UFA. Factors that were protective against all outcomes included older maternal age and more frequent ANC attendance. Additonal protective factors included male gender for LBW and UFA and breastfeeding for UFA.

Our finding that HEU infants have more adverse birth outcomes than HUU infants has been reported in other African studies (Supplementary Table 2) [24]. Within SA, data from a hospital-based cohort study in the Western Cape showed a higher prevalence of PTD and LBW, but not SGA, in HIV-exposed versus HUU infants [25]. Surveillance pregnancy registry data in KwaZulu-Natal also showed a higher prevalence of LBW in HIV-exposed than HUU infants [26]. Another cohort study in the same region reported a higher prevalence of SGA but not PTD in HIV-exposed infants [7]. The mechanisms through which maternal HIV infection results in specific adverse birth outcomes in HEU infants are still unclear. Current evidence suggests that HIV exposure elicits chronic immune activation and systemic inflammation in HEU infants [27, 28], particularly in infants born to women with higher viral loads [29], which have been associated with PTD [30, 31] and LBW [31]. These proposed mechanisms may partly explain why, similar to other studies [32, 33], our study infants born to women with untreated HIV infection had higher rates of PTD compared with infants born to women who initiated ART during pregnancy. These results support the current “test and treat” strategy of initiating all newly diagnosed HIV-positive pregnant women on ART.

However, exposure to ART during pregnancy, a critical period of fetal growth and development, may carry some risks. Evidence on the effect of in utero ART exposure on adverse outcomes is mixed (Supplementary Table 2) [34], and there is still uncertainty as to whether observed adverse effects are specific to particular drugs or combinations thereof. Moreover, as more countries adopt the Option-B+ strategy, many more women will be on ART drugs at conception, highlighting the importance of setting up pregnancy registers or similar surveillance systems to monitor whether earlier ART initiation adversely affects birth outcomes in order to inform future policy. Such monitoring fits into the broader child health goals of reducing child mortality while optimizing good health and well-being [35]. Consistent with findings from a recent review [36], our data show that PTD is higher among women who initiated ART preconception than among mothers initiating ART postconception. These findings are in contrast to the null associations reported in single-site hospital-based retropective [37] and prospective [25] cohort studies conducted in SA and could be biased by the unmeasured maternal disease severity or could be due to selection bias [38]. In line with these studies, however, LBW and SGA did not differ by timing of ART initiation in our sample.

The success of PMTCT programs make it possible for most HIV-exposed infants to remain uninfected [1]. Therefore, while the proportion of under-5 deaths attributed to HIV infection has decreased in SA, the proportion of under-5 deaths due to neonatal conditions has increased. Given that PTD complications constitute the bulk of these neonatal conditions, it is important that risk factors for PTD are addressed through better care of both mothers and infants [39, 40]. Our findings that older maternal age and more frequent ANC visits were protective against all adverse study outcomes, and that infants born to women with lower maternal education and SES had a greater risk for PTD, LBW, and UFA, highlight the importance of health system strengthening and the need for further investment in multisectoral “nutrition-sensitive” interventions [41] that address the multifactorial etiology of these outcomes. The protective effect of breastfeeding on childhood UFA further emphasizes the importance of supporting early initiation of breastfeeding for all infants.

Our study describes birth outcomes in infants that survived their first month of life; therefore, our overall LBW rate is lower than the national estimate of 14.8%, which is based on data from all live births [42]. Background national estimates of PTD and SGA are not readily available in South Africa. Our PTD rate was higher than the 8.0% modeled by Blencowe et al. [43] based on data from 2 hospital-based studies, while our SGA rate is lower than the 23% and 21.8% reported by Lee et al. [44] among preterm- and term-born infants, respectively.

This study has some limitations. First, we were limited by the lack of key clinical data, such as maternal obstetric history, substance use, accurate CD4 cell count, HIV viral load, and whether or not the PTD was induced or spontaneous. While data collectors used a chart to help HIV-infected mothers recall their self-reported ARV drug use, which is subject to recall bias and may cause misclassification of participants, the lack of detailed information on the specific drug and dose used precluded the extent to which study outcomes could be assessed by ART regimen. However, data do show that there is no significant difference in PTD between infants exposed to TDF-containing ART and other combinations [45] although a greater risk of PTD has been reported with protease-inhibitor compared with nucleoside-reverse-transcriptase-based ART [46, 47]. Furthermore, some women initiated on ART precenception may have been on different ART regimens, probably second-line treatment due to drug resistance or poor adherance, than women started postconcepton. Although self-reported CD4 cell count data were also available, we did not include them in the final analysis as the exact timing of the CD4 count testing was unknown, many mothers did not know their results, and those who had the information only reported 1 result. The lack of CD4 cell count and viral load data precluded the extent to which the effect of maternal immune suppression and viremia, respectively, could be assessed. We also could not stratify the ARV analyses by CD4 categories in order to minimize bias by indication. Second, as this was an observational study, we could not establish causal relationships. Nevertheless, in an effort to minimize bias, we included, based on a conceptual framework, factors known to influence our outcomes in the multivariable analyses. Third, we obtained gestational age from the infant’s health card, which contained LMP-based gestational age data. While this method remains the most commonly used method in SA, it has several limitations, including poor recall of the date of LMP and a tendancy to overestimate PTD [48]. However, evidence suggests that LMP is a fairly reliable measure of gestational age in resource-limited settings [49]. We also used routinely collected infant weight data, which are subject to measurement error. Fourth, in accordance with the 2010 WHO PMTCT guidelines, HIV-positive pregnant women who were not eligible for ART were started on ZDV from 14 weeks of gestation to ensure sufficient drug exposure time by late pregnancy [50] as this period carries the highest risk of MTCT [51]. Eligible HIV-positive pregnant women were, however, started on ART immediately in order to improve maternal health and reduce MTCT. We were therefore concerned about potential lead time bias when comparing the outcomes of the ZDV versus ART HEU infants. Although an analysis of our data revealed that the time of exposure to the drugs was similar between the ARV groups, we restricted the ZDV versus ART comparisons to women who initiated ART postconception to minimize this bias. Last, selection bias is possible as sick infants needing emergency care, infants who died before the 6-week clinic visit, and infants attending small remote facilities were excluded. As these infants represent particularly vulnerable groups, our estimates of LBW, SGA, PTD, and UFA could be underestimated.

Notwithstanding these limitations, our study has several strengths. First, this is the largest nationally representitve study of birth outcomes and growth to date among HEU and HUU infants in SA, although it has inherent cross-sectional study limitations related to temporality. Second, the availability of laboratory HIV-1 enzyme-linked immunosorbent assay and PCR results enabled us to exclude HIV-infected infants from the analyses, although NVP exposure may cause false-negative results [52]. Third, we collected data, although not exhaustive data, on maternal, infant, and health system characteristics, which enabled us to explore the independent effect of these factors and adjust for them as potential confounders. Fourth, we estimated birthweight-for-gestational age z scores using the recent Intergrowth standard for term- and preterm-born infants. Last, we collected data before the wide-scale implementation of Option-B+ in SA, which enabled us to compare outcomes of in utero ZDV-exposed versus ART-exposed infants.

In conclusion, there was an association between preconception ART and PTD. As ART access increases, pregnancy registers or similar routine surveillance should be in place to monitor outcomes to inform future policy.

## Supplementary Data

Supplementary materials are available at Open Forum Infectious Diseases online. Consisting of data provided by the authors to benefit the reader, the posted materials are not copyedited and are the sole responsibility of the authors, so questions or comments should be addressed to the corresponding author.

## Supplementary Material

ofx187_suppl_Supplementary_Methods_Tables
