# Supplementary material for: In Utero ART Exposure and Birth and Early Growth Outcomes Among HIV-Exposed Uninfected Infants Attending Immunization Services: Results From National PMTCT Surveillance, South Africa
Source: Open Forum Infect Dis. 2017 Aug 30;4(4):ofx187. doi: 10.1093/ofid/ofx187 (PMC5641411; doi:10.1093/ofid/ofx187)
Supplement: ofx187_suppl_Supplementary_Methods_Tables [file ofx187_suppl_supplementary_methods_tables.docx]

**Supplementary material**

**Sampling**

580 primary health clinics (PHCs) and community health centres (CHCs) offering immunisations services were stratified into 3 strata, within each province, based on their annual 6 week immunisation coverage: small (<130 annual immunisations); medium (130-300 annual immunisations) and large (>300 annual immunisations) facilities. Small facilities were excluded from the final sample due to logistical constraints. Using the 2008 national antenatal maternal HIV prevalence (29.0%) as a cut-off point, facilities in the large immunisation stratum were further stratified to below or above the national average for antenatal HIV prevalence generating a total of 23 strata. A stratified two-stage sampling approach then followed. In the first stage, the primary sampling units (health facilities) were randomly selected proportional to the size of each stratum. In the second stage, a fixed number of infants, comprising of the median number of infants expected within a stratum in a sampling window of 3-4 weeks, was consecutively or randomly selected in each facility. Based on a design effect of 2 and relative precisions for MTCT risk of 30-50% across provinces, a target sample size of 12200 was estimated to generate valid provincial and national estimates for vertical HIV transmission at 6 weeks postpartum [1].

**Laboratory testing**

### Infant dried blood spot (iDBS) specimens were collected by trained nurse data collectors and sent to the National Institute for Communicable Disease (NICD), National Health Laboratory Services (NHLS) for HIV antibody serology testing and, for antibody positive samples, testing for both HIV-1 proviral DNA and HIV-1 RNA using an enzyme immunoassay (EIA) [Genscreen HIV1/2 Ab EIA Version2, Bio-rad Laboratories, France]. Antibody positive specimens collected at the 6week study visit, which underwent a second confirmatory test [Vironostika HIV Uni-form II plus O, bioMérieux Clinical Diagnostics, Marcy-L’Etoile, France], were tested for both HIV-1 proviral DNA and HIV-1 RNA using a qualitative Polymerase Chain Reaction (PCR) COBAS AmpliPrep/COBAS TaqMan (CAP/CTM) Qualitative assay version 1.0, Roche Diagnostics, Branchburg, NJ] to determine infection status. Ten percent of antibody negative tests were re-tested to confirm negativity.

**Construction of socio economic status variable**

Given that our asset variables were discrete we used multiple correspondence analysis (MCA) rather than the principal components analysis (PCA) method [2], which is better suited for normally distributed continuous variables, to construct our socio-economic index [3] using binary variables on household asset ownership (television, refrigerator, stove, phone and car), facilities (access to piped water, electricity and flush toilet) and housing characteristics (brick or non-brick structure).These variables were checked for internal consistency (Cronbach’s alpha=0.73). Using index scores from the first dimension, which explained 84.5% of the variation, we classified households into quintiles: quintile one representing the poorest households and five the least poor. The reliability of the final index was assessed by checking the distribution of certain variables (access to a refrigerator, stove, television, telephone, car, flush toilet and electricity, housing type and mother’s employment status) across the quintiles [4].

**Handling of missing data**

Twenty nine percent of the study participants did not have gestational age information largely because the data were not recorded in their RtHB. As a complete case analysis may introduce bias, we used the inverse probability weighting method to account for the missing gestational age data in the preterm delivery (PTD) and small for gestational age (SGA) analyses. First we generated a predictive logistic regression model for the probability that an individual is missing the binary outcome variable, preterm delivery. The following “fully observed”[5] factors were considered in the model: maternal education and HIV status, child gender and race, household socioeconomic status and an indicator variable for presence of a road-to-health booklet. The goodness-of-fit of the model was then assessed using the Hosmer Lemeshow test [6]. The final weight for the PTD and SGA analyses was estimated as a product of the survey weight and the inverse probability that an individual has missing data for PTD. Only the survey weight was applied to the low birth weight (LBW) and underweight-for-age (UFA) analyses.

Diarrhoea

Low maternal education

Race

HH SES

Maternal age

UFA

TB infection

Syphilis infection

HH food insecurity

Parity

ANC attendance

in-utero ART exposure

LBW

PTD

SGA

Breast feeding

Delivery by C/S

HIV infection

Supplementary figure 1: Simplified conceptual framework of factors associated with birth outcomes and childhood underweight. Note: The associations depicted in the figure are not necessarily uni-directional and possible statistical interactions are not displayed. Abbreviations: ANC; antenatal clinic, ART; antiretroviral treatment, HH; house hold, LBW; low birth weight, PTD; preterm delivery, SGA; small-for-gestational age, Underweight-for-age; UFA

**Supplementary table 1: Prevalence estimates**

| **Outcome** | **Numerator** | **Denominator** |
| --- | --- | --- |
| PTD | Number of infants with gestational age<37 weeks | Number of infants with gestational age data |
| LBW | Number of infants with birth weight <2.5Kg | Number of infants with birth weight data |
| SGA | Number of infants with small for gestational age Z-score <-1.28 | Number of infants with small for gestational age Z-score data |
| UFA | Number of infants with weight-for-age Z-score <-2 | Number of infants with weight-for-age Z-score data |
| Note: Weights were applied to all prevalence estimates. Definitions: low birthweight; LBW, preterm delivery; PTD, small-for-gestational age; SGA, underweight-for-age; UFA, | | |

**Supplementary table 2: Studies comparing birth outcomes in HIV exposed and unexposed African infants**

| Study; design | Setting and data collection period | Adverse birth outcome | PTD  % | LBW  % | SGA  % | Measure of association |
| --- | --- | --- | --- | --- | --- | --- |
| Aniji 2013;[7]  Hospital based retrospective cohort study | South Africa (Johannesburg); Oct2008- Mar2009 | Pre-conception ART | 21 | 21 | ---- |  |
|  |  | Post-conception ART | 24 | 25 | ---- |  |
| Chen 2012[8]; Hospital-based record reviews | Botswana; May2009-April2011 | Overall | 19.6 | ---- | 13.5 |  |
|  |  |  | 23.7 |  | 18.4 |  |
|  |  |  | 17.2 |  | 11.5 |  |
| Ekouevi 2008[9]; Birth cohort | Côte d’Ivoire (Abidjan); Mar 2001-Jul2003 | ART | ---- | HIV exposed | ---- |  |
|  |  | ZDV | ---- | HIV unexposed | ---- |  |
| Ekouevi 2011[10]; Facility based retrospective cohort | Côte d’Ivoire (Abidjan); 2003-2009 | EFV based ART | 9.5 | 17.2 | ---- |  |
|  |  | NVP based ART | 12.7 | 24.2 | ---- |  |
| Fowler 2016[11]; Randomised controlled trial | India, Malawi, South Africa,Tanzania, Uganda, Zambia & Zimbabwe; period 1(April2011-Sep2012); period 2(Oct2012-Oct2014) | ZDV-based ART: Period1 | 20.5 | 23.0 |  |  |
|  |  | TDF-based ART |  | 16.9 | ---- |  |
|  |  | ZDV | 13.1 | 12.0 | ---- |  |
|  |  | ZDV-based ART: Period2 | 19.7 | 20.4 | ---- |  |
|  |  | TDF-based ART | 18.5 | 16.9 | ---- |  |
|  |  | ZDV | 13.5 | 8.9 | ---- |  |
| Kesho Bora Study Group 2011[12]; randomised controlled trial in antenatal clinics | Burkina Faso, Kenya, South Africa. Jun2005-Aug2008 | ART | 13.0 | 11.0 | ---- |  |
|  |  | ZDV & NVP | 11.0 | 7.0 | ---- |  |
| Koss 2014[13]; Hospital based open-label single-site RCT | Uganda (Tororo); 2009-2013 | LPV/r based ART | 16.2 | ---- | ---- |  |
|  |  | EFV based ART | 14.7 | ---- | ---- |  |
| Liu 2014[14]; antiretroviral drug safety study in hospitals and primary health care facilities | South Africa and Zambia; Oct2010-Apr2011 | ART: South Africa | 18.4 | 15.8 | ---- |  |
|  |  | ART: Zambia | 29.7 | 18.1 | ---- |  |
| Li 2016[15]; Clinic-based prospective observational study | Tanzania (Dar es Salaam); Nov2004- Sep2011 | No ARVs | 39 | 17 | 17 |  |
|  |  | All ARV exposed | 29 | 18 | 21 |  |
|  |  | ZDV | 27 | 15 | 9 |  |
|  |  | Pre-conception ART | 38 | 26 | 16 |  |
|  |  | Post-conception ART | 26 | 21 | 15 |  |
| Malaba 2016[16]; Hospital based prospective cohort study | South Africa (Gugulethu); Apr2013-Aug2015 | HIV exposed | 22.0 | 14.0 | 11.0 |  |
|  |  | HUU | 13.0 | 9.0 | 9.0 |  |
|  |  | pre-conception ART | 24.0 | 15.0 | 11.0 |  |
|  |  | post-conception ART | 25.0 | 14.0 | 12.0 |  |
| Marazzi 2011[17]; retrospective cohort study | Malawi and Mozambique; Jul2005-Dec2009 | Overall | 19.1 | 11.5 | ---- |  |
|  |  | No ART | 70 | ---- | ---- |  |
|  |  | ART | 8.5 | ---- | ---- |  |
| Mehta 2017[18]; Pregnancy registry surveillance study | South Africa (Kwa-zulu Natal); Oct2013-Oct2014 | Overall | ---- | 12.4 | ---- |  |
|  |  | HIV exposed | ---- | 14.5 | ---- |  |
|  |  | HIV unexposed | ---- | 11.0 | ---- |  |
| Moodley 2016[19]; Hospital based retrospective record review | South Africa (Kwa-Zulu Natal); Jul-Dec2011 & Jan-Jul2014 | Overall: 2011 | 22.0 | 12.0 | 7.2 |  |
|  |  | Overall: 2014 | 19.5 | 12.8 | 8.2 |  |
|  |  | No ARVs | 32.4 | 23.0 | 10.1 |  |
|  |  | ZDV/NVP | 20.1 | 10.0 | 7.5 |  |
|  |  | NVP based ART | 24.5 | 15.3 | 9.2 |  |
|  |  | EFV based ART | 21.1 | 13.5 | 8.0 |  |
| Ndirandu 2012[20]; Non-randomised antenatal clinic-based intervention cohort | South Africa (Kwa-Zulu Natal) 2001-2004 | Overall | 21.4 | ---- | 16.6 |  |
|  |  | HUU | 21.8 | ---- | 15.1 |  |
|  |  | HEU | 20.9 | ---- | 18.1 |  |
| Nlend 2014[21]; hospital based cross-sectional study | Cameroon (Yaounde) 2008-2013 | Overall | 9.7 | 11.6 | ---- |  |
|  |  | Pre-conception ART | 8.1 | 11.7 | ---- |  |
|  |  | During pregnancy | 10.1 | 11.6 | ---- |  |
| Powis 2011[22]; randomised controlled trial | Botswana; enrolment between Jul2006-May2008 | LPV-based ART | 21.4 | ---- | ---- |  |
|  |  | ABC-based ART | 11.8 | ---- | ---- |  |
| Rempis 2017; cross-section study[23] | Uganda (Fort Portal); Feb-Dec2013 | HUU | 28.2 | ---- | 11.3 |  |
|  |  | Pre-conception ART | 28.9 | ---- | 8.3 |  |
|  |  | Post-conception ART | 27.6 | ---- | 13.8 |  |
| Sofeu 2015[24]; hospital based cohort study | Cameroon; 2007-2010 | HUU | ---- | 4.6 | 3.5 |  |
|  |  | HEU | ---- | 7.5 | 6.3 |  |
| Taha 2012[25]; Six prospective cohort studies | Malawi (Blantyre); 1989-1994; 2000-2007 | HIV exposed | 7.6 | 12.9 |  |  |
| Turner 2013[26]; prospective cohort study | Malawi (Blantyre); 2000-2004 |  |  |  |  |  |
|  |  | HIV exposed | 16.0 | 21.0 | ---- |  |
|  |  | Peripheral viral load increase & low birth weight |  |  |  | PR=1.4 |
|  |  | Placental viral load increase & low birth weight |  |  |  | PR=1.2 |
|  |  | Placental viral load increase & preterm delivery |  |  |  | PR=1.3 |
| Young 2012[27]; prospective clinical trial | Uganda (Tororo); 2009-2013 | HEU | 17.7 | 19.6 | 15.1 |  |
| Zash 2016[28]; hospital-based record review | Botswana (Gaborone & Francistown); May2009-April2011; April2013-April2014 | Overall | 21 | ---- | 18 |  |
|  |  | Initiated EFV-based ART in pregnancy | 18.2 | ---- | 11.9 |  |
|  |  | Initiated other ART in pregnancy | 20.7 | ---- | 21.1 |  |
|  |  | Initiated ZDV in pregnancy | 16.4 | ---- | 20.9 |  |
|  |  | EFV-based ART at conception | 28.0 | ---- | ---- |  |
|  |  | Other ART at conception | 31 | ---- | ---- |  |
| Abbreviations: ABC**;** Abacavir**,**  ARV; antiretroviral, ART: antiretroviral treatment, EFV; Efavirenz, HEU; HIV exposed uninfected, HUU; HIV unexposed, LPV/r; Lopinavir/Ritonavir, low birthweight; LBW, preterm delivery; PTD, small-for-gestational age; SGA, NVP; Nevirapine, ZDV; Zidovudine, underweight-for-age; UFA | | | | | | |

**Supplementary references**

1. Goga AE, Jackson DJ, Singh M, Lombard C, for the SAPMCTE study group. Early (4-8 weeks postpartum) Population-level Effectiveness of WHO PMTCT Option A, South Africa, 2012-2013: South African Medical Research Council and National Department of Health of South Africa, **2015**.

2. Filmer D, Pritchett LH. Estimating wealth effects without expenditure data--or tears: an application to educational enrollments in states of India. Demography **2001**; 38:115-32.

3. Booysen F, Van der Berg S, Burger R, Von Maltitz M. Using an asset index to assess trends in poverty in seven sub-saharan countries World Development **2008**; 36:1113-30.

4. Vyas S, Kumaranayake L. Constructing socio-economic status indices: how to use principal components analysis. Health Policy Plan **2006**; 21:459-68.

5. Seaman SR, White IR. Review of inverse probability weighting for dealing with missing data. Stat Methods Med Res **2013**; 22:278-95.

6. Hosmer DW, Lemeshow S. Applied logistic regression. New York: Wiley, **1989**.

7. Aniji CD, Towobola OA, Hoque ME, Mashamba TJ, Monokoane S. Impact of antiretroviral therapy on pregnancy outcomes. South Afrcian Journal of HIV Medicine **2013**; 14:176-8.

8. Chen JY, Ribaudo HJ, Souda S, et al. Highly active antiretroviral therapy and adverse birth outcomes among HIV-infected women in Botswana. The Journal of infectious diseases **2012**; 206:1695-705.

9. Ekouevi DK, Coffie PA, Becquet R, et al. Antiretroviral therapy in pregnant women with advanced HIV disease and pregnancy outcomes in Abidjan, Cote d'Ivoire. AIDS **2008**; 22:1815-20.

10. Ekouevi DK, Coffie PA, Ouattara E, et al. Pregnancy outcomes in women exposed to efavirenz and nevirapine: an appraisal of the IeDEA West Africa and ANRS Databases, Abidjan, Cote d'Ivoire. J Acquir Immune Defic Syndr **2011**; 56:183-7.

11. Fowler MG, Qin M, Fiscus SA, et al. Benefits and Risks of Antiretroviral Therapy for Perinatal HIV Prevention. The New England journal of medicine **2016**; 375:1726-37.

12. Kesho Bora Study G, de Vincenzi I. Triple antiretroviral compared with zidovudine and single-dose nevirapine prophylaxis during pregnancy and breastfeeding for prevention of mother-to-child transmission of HIV-1 (Kesho Bora study): a randomised controlled trial. Lancet Infect Dis **2011**; 11:171-80.

13. Koss CA, Natureeba P, Plenty A, et al. Risk factors for preterm birth among HIV-infected pregnant Ugandan women randomized to lopinavir/ritonavir- or efavirenz-based antiretroviral therapy. J Acquir Immune Defic Syndr **2014**; 67:128-35.

14. Liu KC, Farahani M, Mashamba T, et al. Pregnancy outcomes and birth defects from an antiretroviral drug safety study of women in South Africa and Zambia. AIDS **2014**; 28:2259-68.

15. Li N, Sando MM, Spiegelman D, et al. Antiretroviral Therapy in Relation to Birth Outcomes among HIV-infected Women: A Cohort Study. The Journal of infectious diseases **2016**; 213:1057-64.

16. Malaba T, Phillips T, Le Roux S, et al. Timing of ART initiation in pregnancy and birth outcomes in South African Women. In: Western Cape Department of Health Research Day (Cape Town).

17. Marazzi MC, Palombi L, Nielsen-Saines K, et al. Extended antenatal use of triple antiretroviral therapy for prevention of mother-to-child transmission of HIV-1 correlates with favorable pregnancy outcomes. AIDS **2011**; 25:1611-8.

18. Mehta S. Understanding of birth outcomes for mothers and infants report **2017**.

19. Moodley T, Moodley D, Sebitloane M, Maharaj N, Sartorius B. Improved pregnancy outcomes with increasing antiretroviral coverage in South Africa. BMC pregnancy and childbirth **2016**; 16:35.

20. Ndirangu J, Newell ML, Bland RM, Thorne C. Maternal HIV infection associated with small-for-gestational age infants but not preterm births: evidence from rural South Africa. Human reproduction **2012**; 27:1846-56.

21. Nlend AN, Zeudja C, Moyo S, Motaze AN, Therapeutic Committee of Centre Hospitalier d’ESSOS Djoungolo. Birth outcomes in HIV-1-Infected women receiving highly active antiretroviral therapy (HAART) prior to conception versus during pregnancy in Yaoundé, Cameroon. Journal of Antivirals and Antiretrovirals **2014**; 6:135-8.

22. Powis KM, Smeaton L, Ogwu A, et al. Effects of in utero antiretroviral exposure on longitudinal growth of HIV-exposed uninfected infants in Botswana. J Acquir Immune Defic Syndr **2011**; 56:131-8.

23. Rempis EM, Schnack A, Decker S, et al. Option B+ for prevention of vertical HIV transmission has no influence on adverse birth outcomes in a cross-sectional cohort in Western Uganda. BMC pregnancy and childbirth **2017**; 17:82.

24. Sofeu CL, Warszawski J, Ateba Ndongo F, et al. Low birth weight in perinatally HIV-exposed uninfected infants: observations in urban settings in Cameroon. PloS one **2014**; 9:e93554.

25. Taha TE, Dadabhai SS, Rahman MH, Sun J, Kumwenda J, Kumwenda NI. Trends in birth weight and gestational age for infants born to HIV-infected, antiretroviral treatment-naive women in Malawi. Pediatr Infect Dis J **2012**; 31:481-6.

26. Turner AN, Tabbah S, Mwapasa V, et al. Severity of maternal HIV-1 disease is associated with adverse birth outcomes in Malawian women: a cohort study. J Acquir Immune Defic Syndr **2013**; 64:392-9.

27. Young S, Murray K, Mwesigwa J, et al. Maternal nutritional status predicts adverse birth outcomes among HIV-infected rural Ugandan women receiving combination antiretroviral therapy. PloS one **2012**; 7:e41934.

28. Zash R, Souda S, Chen JY, et al. Reassuring Birth Outcomes With Tenofovir/Emtricitabine/Efavirenz Used for Prevention of Mother-to-Child Transmission of HIV in Botswana. J Acquir Immune Defic Syndr **2016**; 71:428-36.
